# Author Correction: Biophysical neural adaptation mechanisms enable artificial neural networks to capture dynamic retinal computation

**DOI:** 10.1038/s41467-025-57762-1

**Published:** 2025-03-14

**Authors:** Saad Idrees, Michael B. Manookin, Fred Rieke, Greg D. Field, Joel Zylberberg

**Affiliations:** 1 Department of Physics and Astronomy, York University, Toronto, ON Canada; 2 Centre for Vision Research, York University, Toronto, ON Canada; 3 Department of Ophthalmology, University of Washington, Seattle, WA USA; 4 Department of Physiology and Biophysics, University of Washington, Seattle, WA USA; 5 Stein Eye Institute, Department of Ophthalmology, University of California, Los Angeles, CA USA; 6 Learning in Machines and Brains Program, Canadian Institute for Advanced Research, Toronto, ON Canada

**Keywords:** Biophysics, Retina, Neural circuits, Machine learning, Network models

Correction to: *Nature Communications* 10.1038/s41467-024-50114-5, published online 16 July 2024

In the version of the article initially published, Figs. 2, 3 and Supplementary Fig. 1 displayed results from models in which the movement of static images used to generate a naturalistic movie was mistakenly flipped along the *x*-axis. In the corrected analysis, the revised figures show better performance for all the models, while maintaining the main conclusion of the affected figures and the overall study.

In the third paragraph of the Results, in the sentence now reading “For an example RGC, this conventional CNN model captured 68% FEV of the recorded (Fig. 2a) response with a median FEV of 68% ± 12% across the population (*N* = 57) of recorded cells (Fig. 3a)”, 68% now replaces “59%” and 68% ± 12% replaces “38% ± 8%”. In the sixth paragraph, in the two sentences now reading “Overall, the photoreceptor–CNN model performed with a median FEV of 81% ± 6% (Fig. 3a). This performance gain is substantial given that the photoreceptor layer enhanced the predictive capability of conventional CNNs by approximately 19% (*p* = 1 × 10^–7^)…”, 81% ± 6 now replaces “49% ± 15%”, while 19% (*p* = 1 × 10^–7^) now replaces “29% (*p* = 0.002)”.

In the third paragraph of the Results “Adaptation in the photoreceptor layer drives performance gain” subsection, in the text now reading “When evaluated on the held-out segment of naturalistic movie data, this model performed very similarly to the conventional CNN model, yielding FEV of 73% ± 8% (Fig. 3b; *p* = 0.12)…”, 73% ± 8% now replaces “37% ± 12%” and *p* = 0.12 replaces “*p* = 0.26”. In the paragraph following, the sentence “The resultant photoreceptor–CNN model trained on the same task as above, exhibited lower performance than its fully-trainable counterpart (Fig. 2c; Fig. 3c, FEV 75% ± 6%, *p* = 3 × 10^–7^ two-sided Wilcoxon signed-rank test, *N* = 57 RGCs), but still outperformed the conventional-CNN model when evaluated on the naturalistic movie dataset” now replaces the original “The resultant photoreceptor–CNN model trained on the same task as above, exhibited performance comparable to its fully-trainable counterpart when evaluated on the naturalistic movie dataset (Fig. 2c; Fig. 3c, FEV 49% ± 10%, *p* = 0.07 two-sided Wilcoxon signed-rank test, *N* = 57 RGCs)”.

Figures 2, 3, Supplementary Fig. 1, and their associated source data files have been replaced in the HTML and PDF versions of the article. For comparison, the original figures are available in the Supplementary Information accompanying this amendment.

**Supplementary information**

Original Figs. 2, 3, Supplementary Fig. 1

## Supplementary information

Original Supplementary Information

