# Supplementary material for: Biophysical neural adaptation mechanisms enable artificial neural networks to capture dynamic retinal computation
Source: Nat Commun. 2024 Jul 16;15:5957. doi: 10.1038/s41467-024-50114-5 (PMC11251147; doi:10.1038/s41467-024-50114-5)
Supplement: Supplementary file 3 — Reporting Summary [file 41467_2024_50114_MOESM3_ESM.pdf]

nature portfolio

Corresponding author(s): Joel Zylberberg
Saad Idrees

Last updated by author(s): Jun 5, 2024

Reporting Summary

Nature Portfolio wishes to improve the reproducibility of the work that we publish. This form provides structure for consistency and transparency in reporting. For further information on Nature Portfolio policies, see our Editorial Policies and the Editorial Policy Checklist.

Statistics

For all statistical analyses, confirm that the following items are present in the figure legend, table legend, main text, or Methods section.

n/a	Confirmed	
☐	☒	The exact sample size (*n*) for each experimental group/condition, given as a discrete number and unit of measurement
☐	☒	A statement on whether measurements were taken from distinct samples or whether the same sample was measured repeatedly
☐	☒	The statistical test(s) used AND whether they are one- or two-sided<br>*Only common tests should be described solely by name; describe more complex techniques in the Methods section.*
☒	☐	A description of all covariates tested
☒	☐	A description of any assumptions or corrections, such as tests of normality and adjustment for multiple comparisons
☐	☒	A full description of the statistical parameters including central tendency (e.g. means) or other basic estimates (e.g. regression coefficient) AND variation (e.g. standard deviation) or associated estimates of uncertainty (e.g. confidence intervals)
☐	☒	For null hypothesis testing, the test statistic (e.g. *F*, *t*, *r*) with confidence intervals, effect sizes, degrees of freedom and *P* value noted<br>*Give P values as exact values whenever suitable.*
☒	☐	For Bayesian analysis, information on the choice of priors and Markov chain Monte Carlo settings
☒	☐	For hierarchical and complex designs, identification of the appropriate level for tests and full reporting of outcomes
☒	☐	Estimates of effect sizes (e.g. Cohen's *d*, Pearson's *r*), indicating how they were calculated

Our web collection on statistics for biologists contains articles on many of the points above.

Software and code

Policy information about availability of computer code

Data collection	Visual stimuli were displayed using publicly available MGL codebase available at: http://gru.stanford.edu/doku.php/mgl/overview.<br>Multielectrode array voltages were acquired with customized Labview software.
Data analysis	Spike sorting was performed with custom Java software and Kilosort version 2.5. Most data analysis was performed with open source code (https://github.com/pillowlab/GLMspiketools).<br>Data for models was preprocessed in Python 3.9<br>Models were implemented in Keras (with Tensorflow 2.14.0 backend) package in Python. All modeling code is available at https://github.com/saadidrees/dynret

For manuscripts utilizing custom algorithms or software that are central to the research but not yet described in published literature, software must be made available to editors and reviewers. We strongly encourage code deposition in a community repository (e.g. GitHub). See the Nature Portfolio guidelines for submitting code & software for further information.

Data

Policy information about availability of data

All manuscripts must include a data availability statement. This statement should provide the following information, where applicable:

- Accession codes, unique identifiers, or web links for publicly available datasets
- A description of any restrictions on data availability
- For clinical datasets or third party data, please ensure that the statement adheres to our policy

The retina electrophysiology data used in this study are available under restricted access as they are part of ongoing investigations. Access can be obtained by contacting the corresponding authors. Processed data underlying each figure are provided in the Source Data file.

Research involving human participants, their data, or biological material

Policy information about studies with human participants or human data. See also policy information about sex, gender (identity/presentation), and sexual orientation and race, ethnicity and racism.

Reporting on sex and gender	n/a
Reporting on race, ethnicity, or other socially relevant groupings	n/a
Population characteristics	n/a
Recruitment	n/a
Ethics oversight	n/a

Note that full information on the approval of the study protocol must also be provided in the manuscript.

Field-specific reporting

Please select the one below that is the best fit for your research. If you are not sure, read the appropriate sections before making your selection.

☒ Life sciences ☐ Behavioural & social sciences ☐ Ecological, evolutionary & environmental sciences

For a reference copy of the document with all sections, see nature.com/documents/nr-reporting-summary-flat.pdf

Life sciences study design

All studies must disclose on these points even when the disclosure is negative.

Sample size	4 retina with 37-57 retinal ganglion cells each. Sample sizes were not predetermined. Statistical tests were applied to compare performance of 2 models trained on the same dataset. Number of ganglion cells modeled depended on the number of cells part of that dataset. This number was >= 37 which is typical of retina electrophysiology experiments. For comparing models, individual datapoints representing each cell is provided.
Data exclusions	No data was excluded
Replication	Electrophysiology data was collected and used from 4 independent experiments. For each dataset, more than 100 variants of the models were trained to finetune the hyperparameters. Model with the best accuracy on a validation dataset were used for further analysis. A model with a particular set of hyperparameters was trained multiple times and often on multiple machines to ensure the results could be replicated thereby ensuring stability of the model
Randomization	Randomization was not relevant to this study because the goal was to compare models on different ganglion cells and light levels.
Blinding	Blinding is not relevant because the models are measured objectively using quantified metrics

Reporting for specific materials, systems and methods

We require information from authors about some types of materials, experimental systems and methods used in many studies. Here, indicate whether each material, system or method listed is relevant to your study. If you are not sure if a list item applies to your research, read the appropriate section before selecting a response.

Materials & experimental systems

n/a	Involved in the study
☒	☐ Antibodies
☒	☐ Eukaryotic cell lines
☒	☐ Palaeontology and archaeology
☐	☒ Animals and other organisms
☒	☐ Clinical data
☒	☐ Dual use research of concern
☒	☐ Plants

Methods

n/a	Involved in the study
☒	☐ ChIP-seq
☒	☐ Flow cytometry
☒	☐ MRI-based neuroimaging

Animals and other research organisms

Policy information about studies involving animals; ARRIVE guidelines recommended for reporting animal research, and Sex and Gender in Research

Laboratory animals	- a 11 year old female primate (Macaca nemestrina)<br>- a 17 year old male primate (Macaca mulatta)<br>- 2 Long-Evans female rates (51-215 days old)
Wild animals	n/a
Reporting on sex	Sex was not considered in this study. No known differences in retina processing of male and female.
Field-collected samples	n/a
Ethics oversight	Institutional Animal Care and Use Committee at Duke University and University of Washington

Note that full information on the approval of the study protocol must also be provided in the manuscript.
