# Supplementary figures and images for: Author Correction: Biophysical neural adaptation mechanisms enable artificial neural networks to capture dynamic retinal computation
Source: Nat Commun. 2025 Mar 14;16:2537. doi: 10.1038/s41467-025-57762-1 (PMC11909215; doi:10.1038/s41467-025-57762-1)

Original Fig. 2

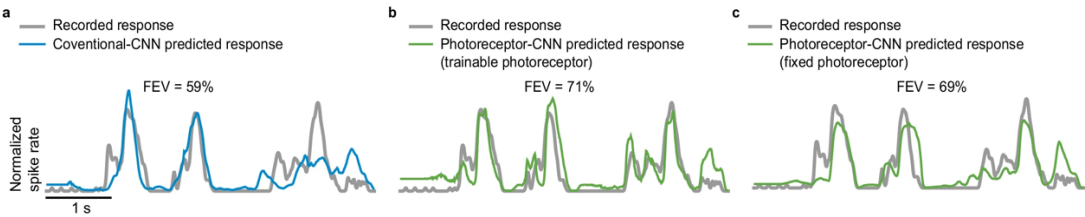

Original Fig. 3

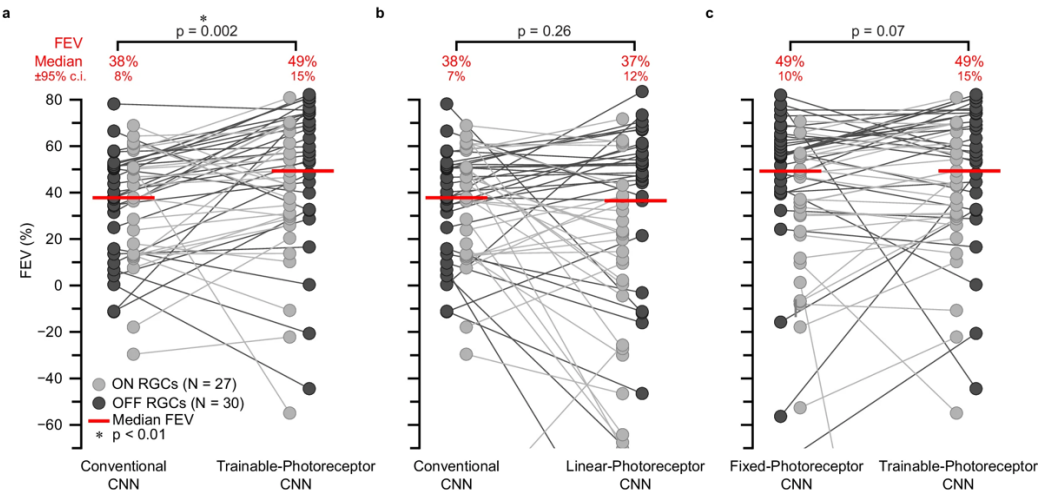

Original Supplementary Fig. 1

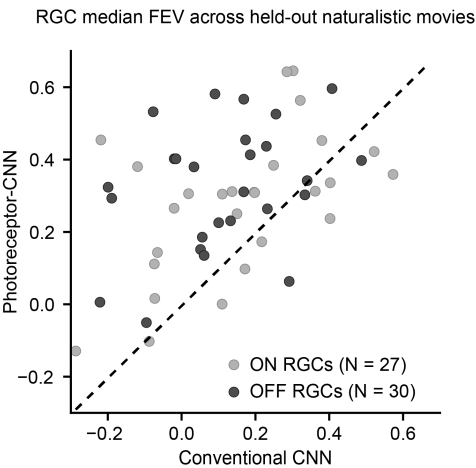

Supplement: Supplementary file 1 — Original Supplementary Information [file 41467_2025_57762_MOESM1_ESM.pdf]
